# Evaluating contributions of urbanization and global climate change to urban land surface temperature change: a case study in Lagos, Nigeria

**DOI:** 10.1038/s41598-022-18193-w

**Published:** 2022-08-19

**Authors:** Liying Guo, Liping Di, Chen Zhang, Li Lin, Fei Chen, Alamin Molla

**Affiliations:** 1 Center for Spatial Information Science and Systems, George Mason University, Fairfax, VA 22030 USA; 2 National Center for Atmospheric Research, Boulder, CO 80301 USA

**Keywords:** Climate sciences, Environmental sciences

## Abstract

This study develops a general method to evaluate the contributions of localized urbanization and global climate change to long-term urban land surface temperature (ULST) change. The method is based on the understanding that long-term annual ULST is controlled by three factors: (1) localized urbanization, (2) global climate change, and (3) interannual climate variation. Then the method removes the interannual climate fluctuations on long-term observed LST time series via linear regression and separates the contributions of urbanization and climate change to the impacts on long-term ULST via urban–rural comparison. The method is applied to Lagos, a fast-growing metropolis in the tropical West Africa, as an example for reference. Combined time-series daily daytime and nighttime MODIS Land Surface Temperature (LST) data over the years of 2003–2021 are used as the representation of land surface temperature. To avoid the potentioal interannual data biase due to uneven availability of data in the rainy seasons over years, only MODIS LST data from dry seasons are used in the study. The results are summarized as follows for Lagos: (1) long-term annual ULST is confirmed to be controlled by the three factors; (2) the proposed method can separate the contribution of the three factors to the ULST; (2) both localized urbanization and global warming are verified to contribute to the ULST increase with positive trends; (3) daytime ULST increased the most in the afternoon time at a mean rate of 1.429 °C per decade, with 0.985 °C (10 year)^−1^ contributed by urbanization and 0.444 °C (10 year)^−1^ contributed by climate warming; (4) nighttime ULST in Lagos increased the most after midnight at a rate of 0.563 °C (10 year)^−1^, with 0.56 °C (10 year)^−1^ contributed by urbanization and 0.003 °C (10 year)^−1^ contributed by climate warming; and (5) urbanization is generally responsible for around 60.97% of the urban warming in Lagos. Therefore, the increasing urbaniztion-induced urban heat island effect is the major cause for more heat-related health risks and climate extremes that many urban residents are suffering. The results of this study are of useful reference for both urbanization and climate change related issues in the geo-science field.

## Introduction

The rapid urbanization in many developing countries over the past decades seems to have been accompanied by extensive land use and land cover changes, which potentially affect local or regional climate through altering the surface energy and water balances^1–6^. One of the major consequences of these modifications is the increase in land surface temperature (LST) in urban areas, which strengthens the urban heat island (UHI) effect^7,8^. The UHI is a localized climate phenomenon whereby urban areas experience warmer temperatures than their surrounding non-urban areas^9,10^. One of the major impacts of the UHI is on microclimates, which are affected by the increased atmospheric temperature within and around urban areas. UHI probably is a driver for the increasing frequency and intensity of extreme weather events in metropolises^11,12^.

The change of surface temperature in urban areas, especially in fast-growing cities, is influenced by three major factors: long-term global climate change, interannual climate fluctuation, and urbanization. Both global climate warming and urbanization tend to increase the temperature in urban areas, while climate fluctuation has either positive or negative impacts on the surface temperature, depending on a specific year. It has been verified that the increase in urban surface temperature is caused by the overlapping impacts of global climate change and localized microclimate change^13^. To understand the urbanization impacts on ULST, the impacts of global changes and interannual climate variation have to be removed from the long-term LST records. However, it is difficult to separate the effects of localized urbanization and global warming on ULST increase from the long-term LST observation records.

The impact of urbanization on urban thermal environments can be assessed by comparing the difference between the urban and rural LSTs^14^. Considerable studies have been conducted in different parts of the world to estimate the temporal change of remotely-sensed LST to determine the UHI effects due to urbanization^15–20^. The satellite remote sensing data, with its large spatiotemporal coverage, is ideal to be used to observe and compare Earth surface changes^21^. Satellite thermal infrared retrievals with continuous estimates have been used to fill gaps in surface air temperature measurements from weather stations^22^. It is well known that observations from satellite remote sensors can only derive under clear-sky weather conditions^23^. However, tropical regions are characterized by high year-round temperatures and seasonal abundant precipitation. It is hard to monitor long-term year-round LSTs in tropical regions from satellite-observed data. Insufficient observations are still a big challenge in estimating the surface meteorological environment.

This study attempts to overcome the above difficulties and challenges in evaluating the urbanization impacts on the ULST change in the context of the global clime change. Lagos, the largest city of Nigeria located in West Africa^21^, is selected as an example of a tropical city in this study. Lagos’s population had risen from 1.4 million in 1970 to 14.9 million in 2021 (https://worldpopulationreview.com/) and is estimated to surpass 88 million by 2100^24^. To increase the temporal satellite-observed coverages, combined daily diurnal (daytime/nighttime) Terra/Aqua MODIS LST products are used over the past two decades in this study. The main work of this study includes (1) validating daily daytime and nighttime Terra/Aqua LST coverages; (2) generating high temporal coverage LST datasets and group time-series annual LSTs; (3) estimating the systematic LST changes by using linear regressions to remove the interannual fluctuations; (4) quantifying the long-term individual contributions from global climate change and localized urbanization to the ULST; (5) and developing a general method to evaluate the UHI effect in the context of climate change.

## Results and discussions

We perform the analysis using the annual dry-season (November, December and January) mean, maximum, and minimum LSTs time series as inputs to estimate the individual contributions of global climate change and localized urbanization to the ULST change.

### Urban study and rural reference sites

**Figure 1:**
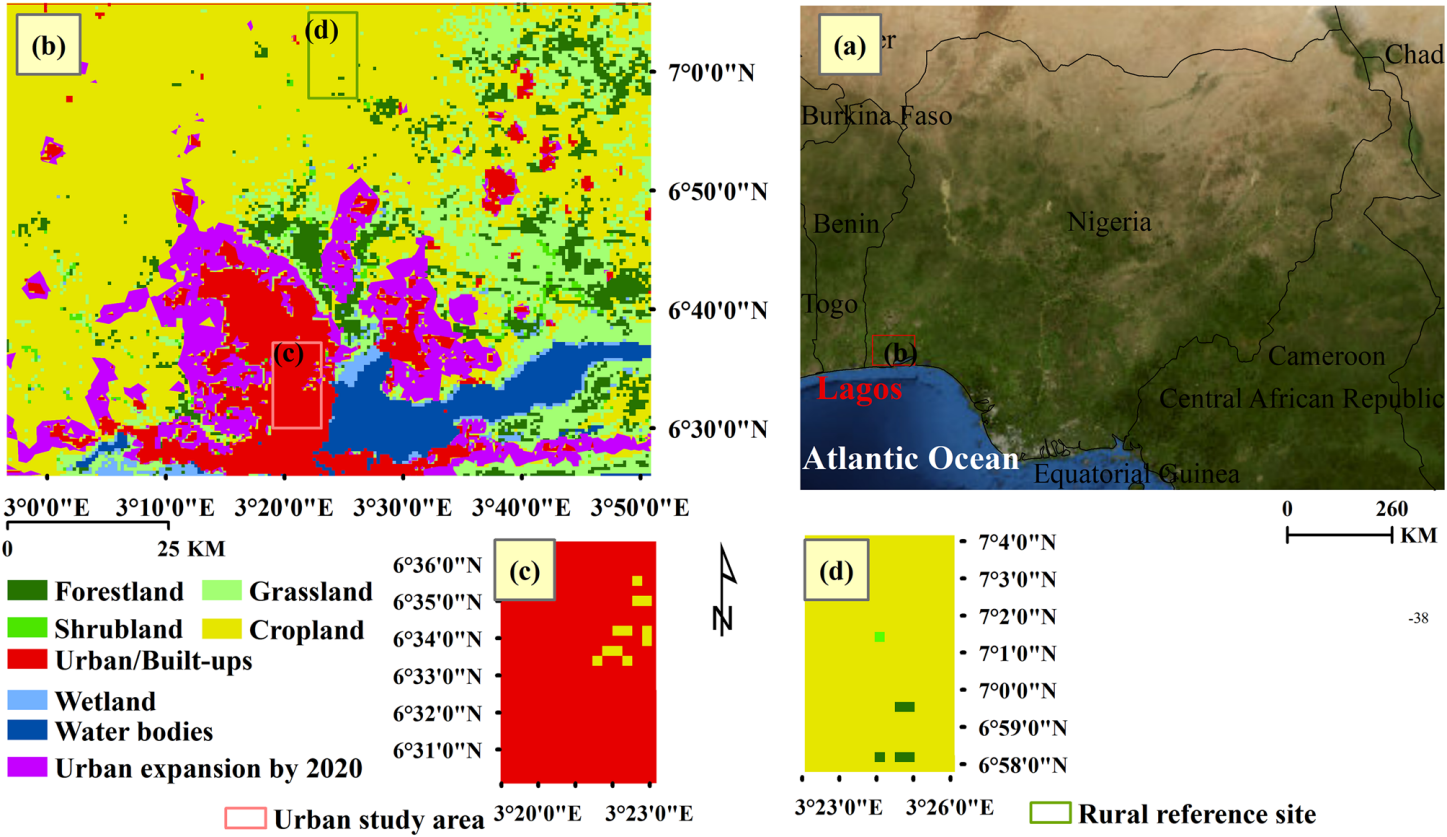
Location of study sites: (**a**) map of Nigeria with Lagos; (**b**) land cover map of Lagos area with both (**c**) urban study area and (**d**) rural reference site based on 2001.

Lagos metropolis as a case study is located in southwestern Nigeria, on the tropical coast of West Africa^25^ (Fig. 1a). It is currently one of the most populated and one of the fast-growing cities in sub-Saharan Africa^26^. The climate in this region is typically tropical with wet and dry seasons. The wet season is from April to October and the dry season is from November to March^27^. The mean annual rainfall is nearly 2000 mm, the minimum air temperatures range averagely from 20.70 to 22.59 °C, and the maximum temperatures from 28.06 to 30.25 °C. But the high temperatures occur usually within the dry season when the average maximum temperatures range from 31.05 to 33.19 °C^28^. The maximum daytime air temperature can reach above 40 °C during the dry season^29^. The highest UHI effects have been observed happening within the dry season and thus pose a higher heat-based health risk to local residents^30^.

The locations of the urbanized area and its reference site are crucial to the estimations. A large Lagos area (Fig. 1b) with both urbanized areas and their suroundings at the size of 105 km × 73.5 km is extracted through the Google Earth Engine (GEE). We delineate a rectangular region within the city center of Lagos totaling 100 km^2^ as an urban study area (Fig. 1c), where land is covered by at least 97% impervious surface based on the land use and land cover (LULC) categories in 2001. The reference site (Fig. 1d) is chosen within a non-urbanized area 30 km away from Lagos city, with the same size and shape as the urban study area. More than 95% of land in the reference site is covered by crop and little land-use conversion took place over the last twenty years. The reference site is selected based on the fact that it is located in a non-urban region with a similar climate zone as urban areas but far away from the impact of urbanization on LST, and its LULC have changed little over the years. In this study, LULC data are retrived from the International Geosphere-Biosphere Programme (IGBP) of MODIS MCD12Q1 product^31,32^, which is an annual global land cover classification product at 500-meter resolution, available from 2001 to 2020. The descriptions of IGBP land cover classes can be found in Liang et al.^33^.

### The annual variations for ULSTs and RLSTs

**Figure 2:**
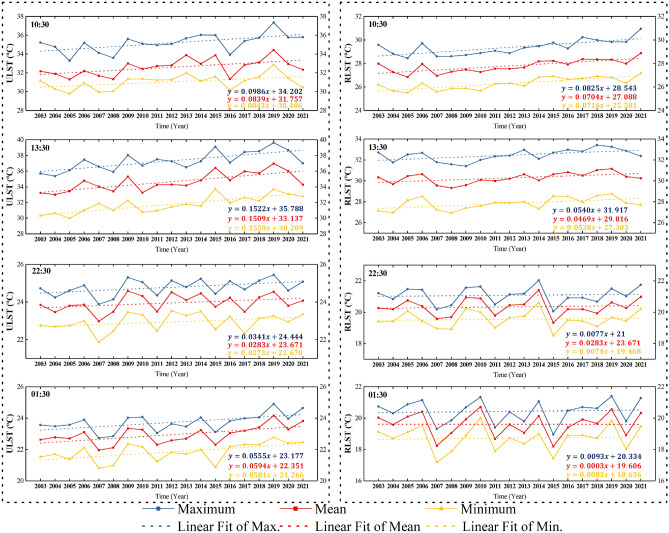
The annual fluctuations and their linear trends with separate linear regression models of the maximum, mean, and minimum LSTs for the period 2003–2021 over the urban area (left plots) and rural area (right plots) in Lagos during four observation times at 10:30, 13:30, 22:30, and 01:30. The blue, red, and yellow solid curves depict respectively the annual variations of the maximum, mean, and minimum LSTs. The straight dot lines represent their linear fits calculated by the linear regression models with the same colors for the max., mean, and min. groups. The equations at the bottom right are their linear regration models, where $y$ means the fitted value of LST and $x$ is the progressive time (year).

**Figure 3:**
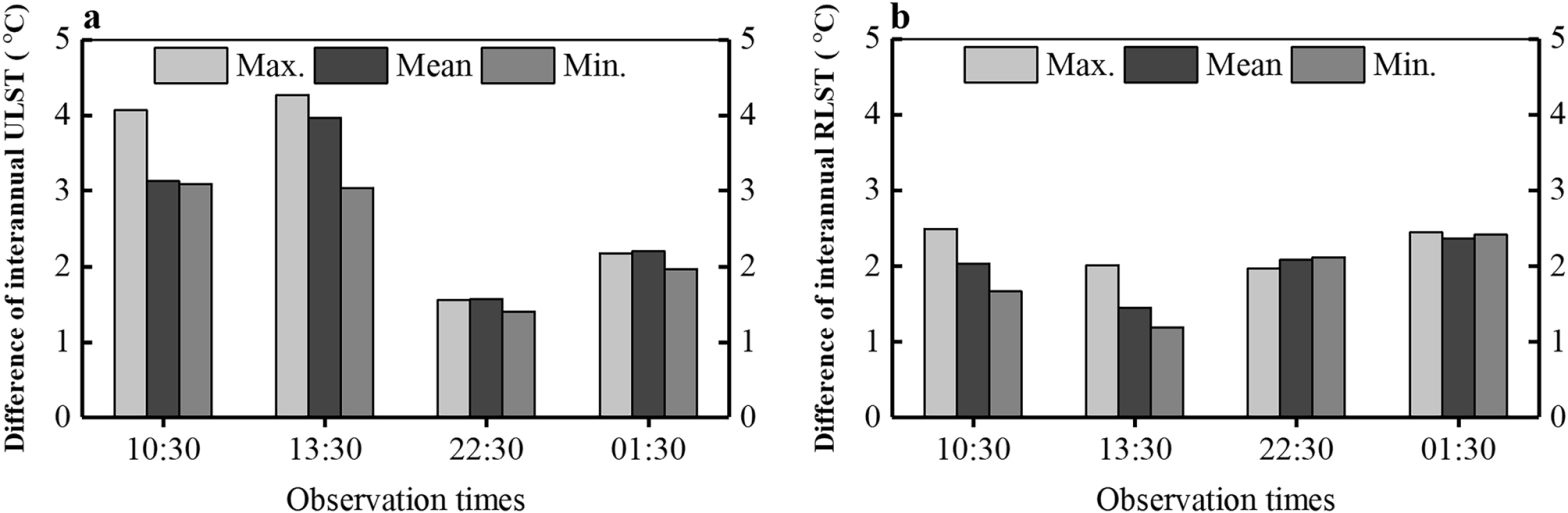
The difference in annual maximum, mean, and minimum LSTs during four observation times at 10:30, 13:30, 22:30, and 01:30 for the urban area (**a**) and rural area (**b**). The values are obtained by calculating the difference between the highest and lowest annual LSTs during the time period in a specific group.

Figure 2 depicts the annual fluctuations and their long-term linear trends as well as the corresponding linear regression models of the maximum, mean, and minimum LSTs for the period 2003–2021 over the urban area (left plots) and rural reference area (right plots) in Lagos during four observations at 10:30, 13:30, 22:30, and 01:30 local times. The results show that all LSTs of time series have the similar linear growth patterns over time. It is also found that there are interannual fluctuations in all long-term LSTs, especially in the urban area. Figure 3 illustrates the largest magnitudes of interannual variations, which are obtained by calculating the difference between the highest and lowest annual LSTs during the time period in a specific group. Generally, ULSTs exhibit the largest interannual fluctuation reaching a maximum of 4 °C difference in the daytime. RLSTs are relatively flat with only around 2 °C difference. The results confirm that the long-term ULSTs during the days have higher interannual variations than those during the nights, while no significant difference can be observed both daytime and nighttime in the RLSTs.

### The temporal trend of LST time series

**Figure 4:**
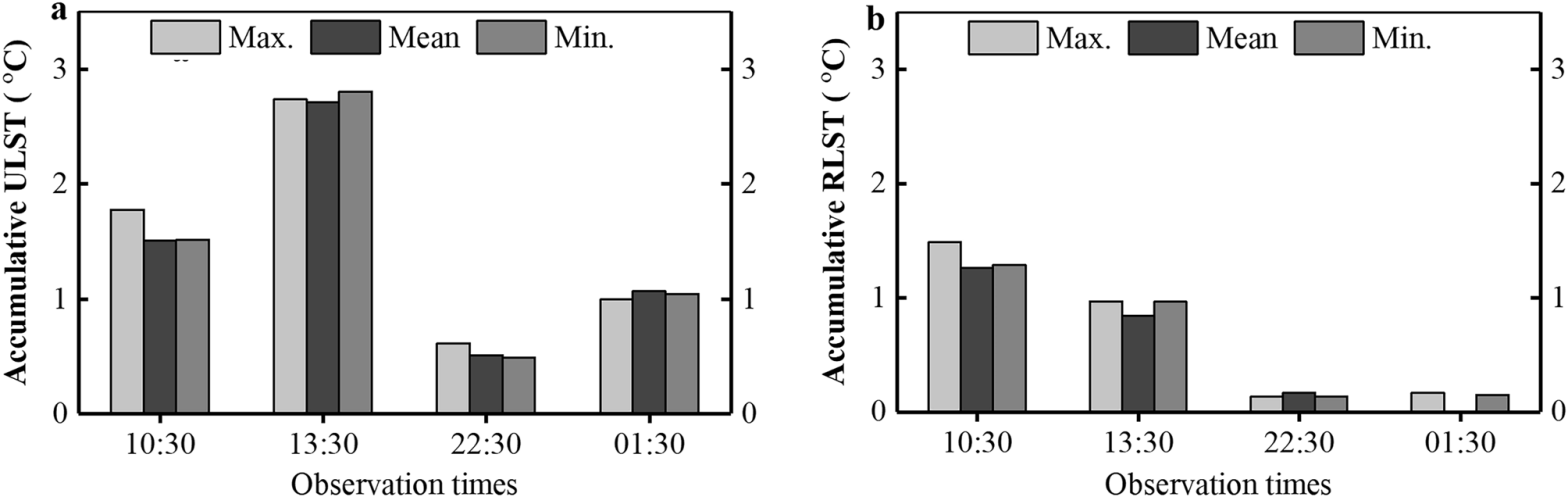
The accumulative maximum, mean, and minimum LSTs during four observation times at 10:30, 13:30, 22:30, and 01:30 for the urban (**a**) and rural area (**b**).

As introduced below in the "Methods" section, the values of ${\Delta T}_{U}$ and ${\Delta T}_{G}$ can be obtained by calculating fitting values from linear regression models for the urban and reference site to remove the effects of the interannual variation and global climate change from the time series, respectively. Therefore, the specific linear regression models are generated by time-series LST groups as shown in Fig. 2. The specific fitted LSTs for all time series are generated with the corresponding linear regression models. From the linear fitting lines, it can be observed that all LSTs have evident positive trends. The total accumulative increases for all LSTs are computed separately from the difference of the fitting value between the final year and the initial year as displayed in Fig. 4. The results further confirm that the warming is noticeable over the time, especially during the daytime. The annual LST in the daytime during the dry season is assessed to exceed 38.68 °C in 2021with an annual maximum warming rate of 0.144 °C. What’s more, urban areas tend to suffer stronger warming than rural areas. During the daytime, the mean ULST has an accumulative warming of 2.113 °C over the 19-year period with an average annual mean warming rate of 0.111 °C. The warming trend is also observed in the rural reference area with 1.056 °C mean accumulative warming. During the nighttime, ULST is with an accumulative warming of 0.789 °C, and RLST is negligible, merely 0.088 °C over the 19-year period.

### The contribution of localized urbanization and global warming to ULST

Table 1 shows the specific values of decadal change rates of annual mean, maximum and minimum LSTs at four observation times. From Table 1, it is clear that both urbanization and climate warming contribute to ULSTs increase. During the daytime, the decadal change rates of ${\Delta T}_{U}$ are 0.795 °C, 0.934 °C, and 0.799 °C at 10:30 AM, and 1.430 °C, 1.442 °C, and 1.477 °C at 1:30 PM for the annual mean, maximum and minimum temperatures, respectively. The change rates of ${\Delta T}_{G}$ is 0.709 °C (10 year)^−1^ at 10:30 AM and around 0.489 °C (10 year)^−1^ at 1:30 PM. During the nighttime, the decadal change rates of ${\Delta T}_{U}$ have a higher increase in annual temperatures at 1:30 AM (around 0.546 °C) than at 10:30 PM (around 0.283 °C), while the warming rates of ${\Delta T}_{G}$ is around 0.078 °C (10 year)^−1^ at 10:30 PM and around 0.057 °C (10 year)^−1^ at 1:30 AM. In general, the annual ULST/RLST changes are all positive during the day and night, and the warming is higher during the day than at night. Comparing values of both ${\Delta T}_{U}$ and ${\Delta T}_{G}$, it can be found that during the day, global warming contributes more to the ULST increase at 10:30 AM than at 1:30 PM.

**Table 1:** The decadal change rates of annual mean, maximum and minimum temperatures at four observation times.

°C (10 year^−1^)	Daytime						Nighttime					
	10:30			13:30			22:30			01:30		
	Max.	Mean	Min.	Max.	Mean	Min.	Max.	Mean	Min.	Max.	Mean	Min.
Δ*T*_*U*_	0.934	0.795	0.799	1.442	1.430	1.477	0.323	0.268	0.259	0.526	0.563	0.550
Δ*T*_*G*_	0.782	0.667	0.678	0.512	0.444	0.510	0.073	0.090	0.070	0.088	0.003	0.079
Δ*T*_*UHI*_	0.153	0.128	0.121	0.930	0.985	0.967	0.250	0.178	0.189	0.438	0.560	0.472

Since the urbanization has little impact on the reference region, the ${\Delta T}$ in the rural area is considered to be approximately the ${\Delta T}_{G}$ in the urban area. With this approximation, the contribution of urban expansion to the change of urban LST ($\Delta {T}_{UHI}$) can be obtained with Eq. (2). The results, as shown in Table 1, indicate that during the daytime urbanization contributes significantly more to urban warming in the afternoon than in the morning, and urbanization is also a dominant contributor to urban warming during the nighttime compared with global warming.

**Figure 5:**
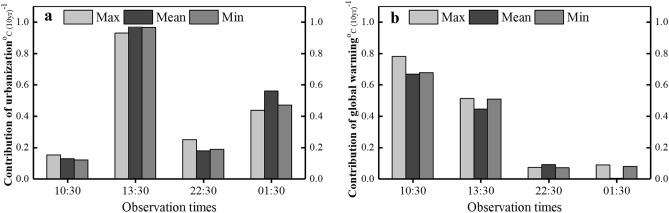
The long-term contributions of urbanization (**a**) and global warming (**b**) at four observation times.

Figure 5 depicts the long-term contributions of urbanization (a) and global warming (b) on the surface temperature around at four observation times. No significant difference can be observed among mean, maximum, and minimum LSTs in their contributions. On the whole, it is estimated that urbanization contributes averagely 60.97% of the urban warming. Unlike the urban study area, which is mainly covered by impervious materials, the rural reference site is covered with crops, grasses, shrubs, and trees, which can convert a lot of incoming solar radiation to latent heat through evapotranspiration. Therefore, it’s easy to understand that the urban LST is consequently higher compared to that in the reference site^25,26,34^. The urban temperature anomaly tends to increase primarily with the changes in city morphology and characteristics of urban surface, such as density and height of buildings, and the materials of both roofing sheets and impervious surface, which also could block more re-radiation from the surface for cooling down at night^13,27^. The rural surfaces are comparatively simple and therefore the thermal radiation emitted experiences greater nocturnal radiative flux divergence than complex urban surfaces^35,36^. It generally follows why urbanization leads to the rise in temperature of the urban areas, and the stronger UHI effect is observed during the afternoon and night. The increasing urban-induced warming could cause urban residents to be uncomfortable and increase the likelihood of extreme events in the future^37^. Therefore, quantifying the impact of urbanization on UHI is of great value for future researches on how to mitigate the UHI effects and enhance the comfort of tropical dwellers living in cities.

## Conclusions

In this study, we developed a general method to quantify the individual contributions of localized urbanization and global warming to ULST increase and applied the method to Lagos as an example. Daily diurnal MODIS LST products from both Terra and Acqua satellites during January, November, and December (dry season) for the period of 2003–2021 are used in the case study. We selected the city center of Lagos as the urban study area and its surrounding non-urban area as the reference site based on the land cover in 2001. The main conclusions are summarized as follows.

1. Long-term annual urban LST is influenced by the combined effects of localized urbanization, global climate change, and interannual climate variation. The systematic analysis is proposed to quantify and confirm their combined impacts on long-term annual dry-season ULSTs. It is of great reference for monitoring the local climate change in the context of urbanization and global climate change.
2. The combination of daytime and nighttime Terra/Aqua MODIS LST products is optimal for producing annual observed LST time series for the past two decades. It fills data biases derived from satellite remote sensing data in the tropics.
3. Linear regressions are established to remove the interannual climate fluctuations on long-term observed LST time series. Analysis indicates that the daytime magnitudes of annual dry-season ULSTs are at the highest interannual fluctuation, reaching beyond a 4 °C difference in maximum temperature and a 3 °C in mean/minimum. The nighttime interannual ULSTs fluctuate from 1.4 to 2.2 °C. In contrast, the interannual fluctuation of RLSTs is generally flat with only around a 2 °C difference.
4. Urban–rural comparison method has been developed to separate the contributions of urbanization and global climate change to long-term ULST impacts. The location of a reference site is crucial to the estimations. Both localized urbanization and global climate change are verified to contribute to the increase in dry-season ULSTs with positive long-term trends during the day and night. The warming is higher during the day than at night. The annual global climate change during the day is observed to have more contribution in the morning than in the afternoon with a maximum of 0.782 °C per decade around 10:30 AM local time.
5. Daytime LST in Lagos increased the most in the afternoon at a mean rate of 0.1429 °C per year over the past two decades, with 0.0985 °C per year contributed by urbanization and 0.0444 °C per year contributed by global climate change. Nighttime LST in Lagos increased the most after midnight at a rate of 0.0563 °C per year, with 0.056 °C per year contributed by urbanization and 0.0003 °C per year contributed by global climate change
6. Generally, urbanization and global climate change contributed averagely 60.97% and 39.03% of the urban warming in Lagos, respectively.
7. Lagos, a typical large tropical metropolis with rapid urban expansion, is ideal for studying the impacts of urbanization and climate changes on LST change. The increasing urban-induced heat in Lagos could cause urban residents to suffer more heat-based health risks and increase the extreme events. Therefore, quantifying the impact of urbanization on UHI is of great value for future researches on how to mitigate the UHI effect and enhance the comfort of tropical dwellers living in cities.

At the end, we would also like to point out the limitations of results from this study. Although the method we developed in this study is general and location-independent and can be applied to any urban areas in the world, the conclusions about warming degrees, the temperature increase rates, and the contribution percentages by the urbanization and global warming are only relevant to Lagos. This is because urbanization speed, characteristics, urban landscape are different from different urban areas and the magnitude of global warming impacts is location dependent. In the future study, we can apply the method to urban areas at different climate zones with different socioeconomic status to find out how these factors can impact the UHI effects.

## Methods

### Intruduction to methods

The main objective of this study is to develop a general method to estimate the contributions of localized urbanization and global climate change on ULSTs and take Lagos as a case study to apply this method for estimations. It is well known that global warming and urbanization are the two key contributors to the higher LST in cities^38^. In addition, the interannual climate fluctuation also plays a role in the annual LSTs. In order to realize the objectives of this study, we have to separate the contributions of these three factors to the annual LSTs in a city.

It can be reasonably assumed that the annual (mean, min., or maximum) LSTs will fluctuate around a constant value due to interannual climate variations if there is no global warming or urbanization. It is also assumed that both global warming and urbanization contribute linearly to the LST increase in urban areas over the years. Therefore, a linear regression of the annual LST time series will remove the contribution of interannual climate variations to the annual LST, and the linear function obtained from regression represents the long-term systematic trend of the LST for a city area, which represents the combined contributions of both the urban expansion and local impact of global warming. Although the trend of temperature increase due to global warming varies globally, the trends for the two nearby areas within the same climate regime should be similar if not the same. That is why we select a nearby area, which has been neither subjected to urbanization nor experienced a large land use/land cover change, as the reference site for quantifying the contribution of global warming. The time series of annual LSTs and the linear regression are also calculated for the reference site. The linear function obtained from the regression on the reference site represents the trend of LST change due to global warming. The trend from the reference site can be used to approximate the trend of LST change due to global climate change in urban areas. Therefore, it can be assumed that the difference in the linear functions between the urban study area and the rural reference site can be attributed to the contribution of urbanization to ULST change.

Thus, the contributions of both localized urbanization and global warming can be quantified by the following steps: (1) calculating annual time-series LSTs for both the urban study area and the reference site; (2) using linear regressions to remove the interannual variation from the time series of both the study area and the reference site. The linear functions resulting from linear regression represent the long-time trends of LSTs in the study area and the reference site, respectively; (3) For the urban study area, calculating the systematic trend of LST contributed by the combination of urban expansion and local impact of global warming as shown in Eq. (1); and (4) deriving LST increase contributed by urbanization through removing the contribution of global warming from the systematic trend of LST in the reference site as shown in Eq. (2).

Based on the discussion above, ($\Delta {T}_{U})$ is the combination of the UHI effect caused by urbanization and the local impact of global warming. Thus,

1 $$\Delta {T}_{U}=\Delta {T}_{UHI}+{\Delta T}_{G}$$

where $\Delta {T}_{U}$ denotes the overall systematic ULST change from the initial year to the final year, $\Delta {T}_{UHI}$ means the LST change due to the urbanization effect, and ${\Delta T}_{G}$ is the overall LST change due to global warming. The values of $\Delta {T}_{UHI}$ thus could be calculated by

2 $$\Delta {T}_{UHI}={\Delta T}_{U}-{\Delta T}_{G}$$

Therefore, to derive $\Delta {T}_{UHI}$, we have to obtain ${\Delta T}_{G}$ first by finding a region that is not only close enough to the city so that the local impact of global warming is similar by being within the same or similar climatic zone, but also far enough from the city so that its LST is not impacted by the urbanization in the city. This region is called the reference site, which is usually selected from the nearby rural areas. Further, this site and its immediate neighbor should have no significant land use and land cover change (LULCC) over a long period of time. As such, the systematic LST change in this site is most likely to be linked to external atmospheric forcing, i.e., global climate change. The annual LST time series for the reference region then is calculated from the MODIS LST products and the linear regression on the time series is performed to remove the interannual variability. Then, ${\Delta T}_{G}$ can be obtained by

3 $${\Delta T}_{G}={T}_{Rf}-{T}_{Ri}$$

where ${T}_{Rf}$ and ${T}_{Ri}$ are the LSTs of the reference region at the final time and the initial time of the time series, respectively, obtained through the linear regression equation.

**Figure 6:**
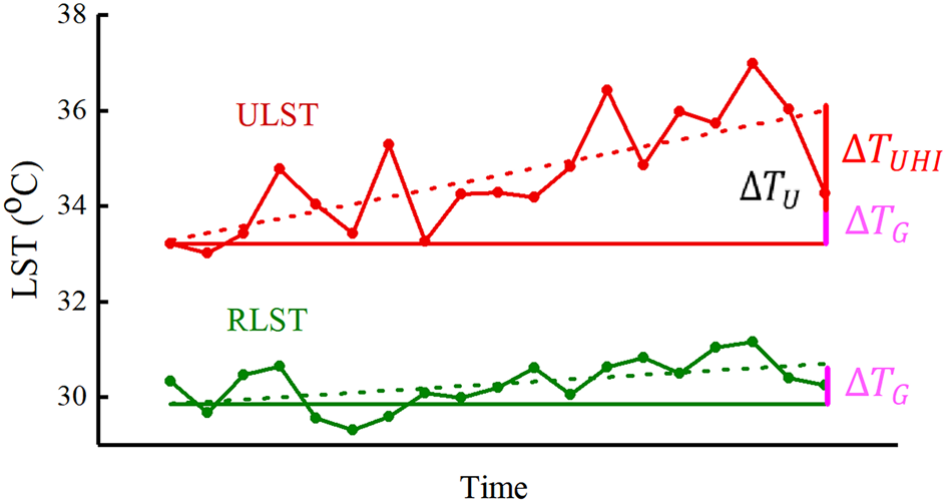
A combined diagrams to depict the methods in this study.

Figure 6 provides the graphic description that explains the method in this study. In the figure, the curves represent the time series of annual specific LSTs for the urban study area (red) and the rural reference area (green). The straight dot lines are the minimum mean square error (MMSE) linear fitting obtained through linear regression as the trends of the LST time series for the urban (red) and rural areas (green). ${\Delta T}_{U}$ (in dark) is the systematic LST change during the study period for the urban area, which is the difference between the fitted value of ULST at the final time of the time series and that value at the initial time of the time series. ${\Delta T}_{G}$(in purple) is the contribution of global climate change to ${\Delta T}_{U}$. ${\Delta T}_{G}$ is calculated by the temperature difference between the fitted value of LST at the final time and that value at the initial time of the time series for the rural area. $\Delta {T}_{UHI}$(in red), the contribution of urbanization to long-term ULST change $\boxtimes$, is calculated by the difference between ${\Delta T}_{U}$ and ${\Delta T}_{G}$.

### LST data acquistition

One effective method to measure LST is thermal remote sensing. In this study, we use two daily MODIS Land Surface Temperature & Emissivity products, MOD11A1 (version 6) and MYD11A1 (version 6), from the National Aeronautics and Space Administration (NASA). The MOD11A1 daily LST product, available from February 24, 2000, is derived from the MODIS sensor onboard the Terra satellite. And the MYD11A1 daily LST product, available from July 04, 2002, is derived from the MODIS sensor onboard the Aqua satellite. Thereby, the MODIS LSTs are compiled from January 01, 2003 to December 30, 2021. Both Terra and Aqua MODIS products provide daily global coverage (http://modis.gsfc.nasa.gov). They have different local overpass times, i.e., Terra descending around 10:30 and ascending around 22:30 at local time, Aqua descending around 01:30 and ascending around 13:30 at local time, which allow the two MODIS sensors to observe the Earth surface four times per day at 01:30, 10:30, 13:30, and 22:30 local time. Clouds and other atmospheric disturbances often obscure parts of or even the entire observation scene, which is a significant obstacle to continuously monitor or predict LST changes, especially in tropical regions^23^. The Terra and Aqua MODIS LST products are only captured under cloud-free conditions^32^. Therefore, data availability of LSTs may influence the accuracy of the accumulated annual LST estimations^23,39^.

Google Earth Engine (GEE) platform is used to access daily LST time series, convert LSTs units, and calculate the regional average LSTs for the urban study area and rural reference site. Specifically, the daily daytime and nighttime LSTs (LST_Day_1km and LST_Night_1km) are selected firstly from MOD11A1 and MYD11A1, respectively. Next, the LST values are converted from Kelvin to Celsius units using the following formula:

4 $${T}_{c}=0.02{T}_{k}-273.15$$

where ${T}_{c}$ is the temperature in Celsius (°C), ${T}_{k}$ is the scaled absolute temperature in Kelvins (K) stored in the MODIS LST products, and 0.02 is a scale factor. And then an existing GEE function is applied to calculate a single cumulative value of the mean/max./min. LSTs during the study time period. Lastly, the boundary polygons that determined the urban study area and rural reference region are uploaded to clip the corresponding LST values and then export these LSTs to Google Drive.

### LST data availability

When deriving meteorological parameters from remote-sensing time series, those satellite observations are expected to be presented in good quality^40^. Due to cloud cover and weather conditions, the remotely sensed LST time-series products, especially in the tropical regions, contain both spatial and temporal gaps and missing values, which could cause undesirable uncertainties in the analysis. To increase the spatial/temporal coverage of LST in the Lagos area, combined daily diurnal MODIS LSTs from both Terra and Aqua satellites are derived in this study to generate the daytime and nighttime LST time series. Because the MYD11A1 product is available from July 2002, which is later than the available date of MOD11A1, the time period of data collection for this study is thus set to the time period from the first day of 2003 to the last day of 2021. Since the entire temporal period covers a total of 6940 days (19 years) as well as four observation times each day, the amount of remotely sensed LST images is huge to be processed and calculated. Therefore, the Google Earth Engine (GEE) (https://earthengine.google.com/platform/), which is a cloud-based platform for a variety of geospatial analyses, is performed to collect and process time-series daily mean, maximum and minimum ULSTs and RLSTs from daytime and nighttime MOD11A1 and MYD11A1 products.

**Figure 7:**
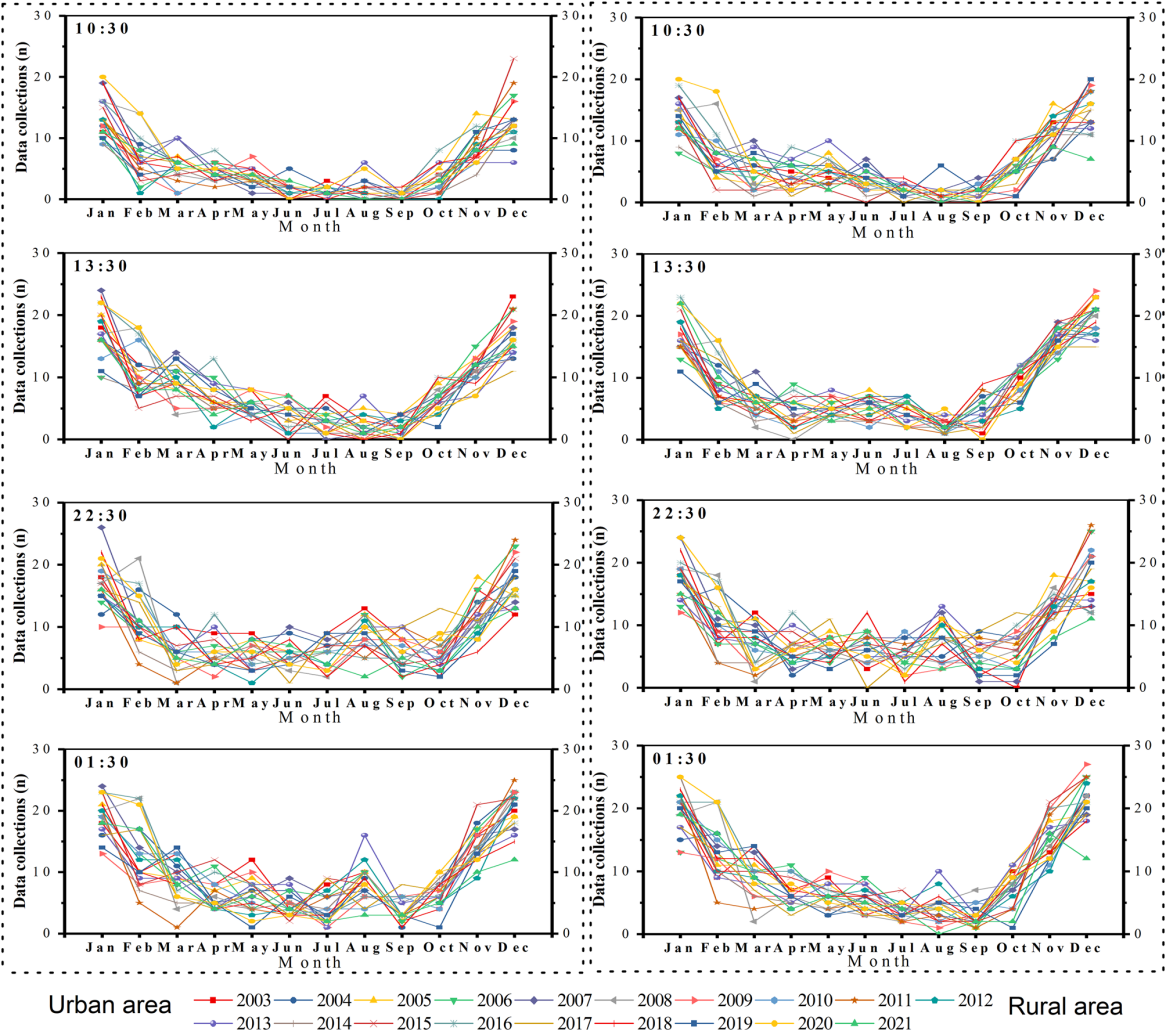
The numbers of data collection by month across the urban area (left plots) and rural reference area (right plots) for the years of 2003 to 2021 at four observation times.

As introduced above, Lagos is located within a tropical climate zone characterized by high year-round temperatures and abundant seasonal precipitation. Due to frequent cloud cover in this region, satellite-observed LST data are not available for each day. Particularly during the wet seasons, LST images are only available for a few days each month. Statistics from LSTs of those few days are hardly representative of the LSTs of the month, which could result in inaccurate LSTs in month or year and further estimations^41^. To verify and validate this issue, we further count the specipic numbers of cloud-free LST images from MODIS at four observation times for each day in the years 2003–2021 for both study sites. Figure 7 depicts the total numbers of LSTs collected at four observation times for each month of the year throughout the study period across the urban area (left plots) and the rural reference site (right plots), respectively. The higher collections can be found in Jannuary, December, and November. Although satellite remote sensing is an excellent data source for monitoring the Earth surface characteristics on a large scale^42^. It is still challenging to retrieve the satellite-observed land surface characteristics in the tropics. Therefore, for this study, we only compile daily time-series LSTs in January, November, and December for the urban and reference sites to calculate the annual dry-season LSTs. For simplicity, we use the annual LSTs to represent the annual dry-season LSTs in this study. The data processing and results acquisition are in accordance with the methods discussed above.

All raw data used in this study, including MOD11A1 (v6), MYD11A1 (v6), and MCD12Q1 (v6), are freely available and online accessible from the following links:

- https://developers.google.com/earth-engine/datasets/catalog/MODIS_061_MOD11A1?hl=en.
- https://developers.google.com/s/results/earth-engine/datasets?hl=en&q=MYD11A1.
- https://developers.google.com/earth-engine/datasets/catalog/MODIS_006_MCD12Q1?hl=en.

## References

[1] Han W (2020). The mechanisms and seasonal differences of the impact of aerosols on daytime surface urban heat island effect. Atmos. Chem. Phys..

[2] Pongratz J (2006). The impact of land cover change on surface energy and water balance in Mato Grosso, Brazil. Earth Interact..

[3] Yu M, Liu Y, Dai Y, Yang A (2013). Impact of urbanization on boundary layer structure in Beijing. Clim. Change.

[4] Guo L, Di L, Tian Q (2019). Detecting spatio-temporal changes of arable land and construction land in the Beijing-Tianjin corridor during 2000–2015. J. Geogr. Sci..

[5] Liu Y, Yan B, Zhou Y (2016). Urbanization, economic growth, and carbon dioxide emissions in China: A panel cointegration and causality analysis. J. Geogr. Sci..

[6] Guo L, Di L, Zhang C, Lin L, Di Y (2022). Influence of urban expansion on lyme disease risk: A case study in the U.S. I-95 northeastern corridor. Cities.

[7] Shahfahad (2020). Longitudinal study of land surface temperature (LST) using mono- and split-window algorithms and its relationship with NDVI and NDBI over selected metro cities of India. Arab. J. Geosci..

[8] Dutta D, Rahman A, Paul SK, Kundu A (2019). Changing pattern of urban landscape and its effect on land surface temperature in and around Delhi. Environ. Monit. Assess..

[9] Zhao L, Lee X, Smith RB, Oleson K (2014). Strong contributions of local background climate to urban heat islands. Nature.

[10] Shastri H, Barik B, Ghosh S, Venkataraman C, Sadavarte P (2017). Flip flop of day-night and summer-winter surface urban heat island intensity in India. Sci. Rep..

[11] O’Malley C, Piroozfarb PAE, Farr ERP, Gates J (2014). An investigation into minimizing urban heat island (UHI) effects: A UK perspective. Energy Procedia..

[12] Doan VQ, Kusaka H (2018). Projections of urban climate in the 2050S in a fast-growing city in Southeast Asia: The Greater Ho Chi Minh City metropolitan area, Vietnam. Int. J. Climatol..

[13] Giridharan R, Emmanuel R (2018). The impact of urban compactness, comfort strategies and energy consumption on tropical urban heat island intensity: A review. Sustain. Cities Soc..

[14] Shahfahad (2021). Urban heat island dynamics in response to land-use/land-cover change in the coastal city of Mumbai. J. Indian Soc. Remote..

[15] Henits L, Mucsi L, Liska CM (2017). Monitoring the changes in impervious surface ratio and urban heat island intensity between 1987 and 2011 in Szeged. Hungary. Environ. Monit. Assess..

[16] Chen T (2021). Mapping temporal and spatial changes in land use and land surface temperature based on MODIS data. Environ. Res..

[17] Mohammad P, Goswami A (2021). A spatio-temporal assessment and prediction of surface urban heat island intensity using multiple linear regression techniques over Ahmedabad City, Gujarat. J. Indian Soc. Remote.

[18] Sharma R, Pradhan L, Kumari M, Bhattacharya P (2021). Assessing urban heat islands and thermal comfort in Noida city using geospatial technology. Urban Clim..

[19] Xing Z (2021). Estimation of daily mean land surface temperature at global scale using pairs of daytime and nighttime MODIS instantaneous observations. ISPRS J. Photogramm..

[20] Imhoff ML, Zhang P, Wolfe RE, Bounoua L (2010). Remote sensing of the urban heat island effect across biomes in the continental USA. Remote Sens. Environ..

[21] Crowley MA, Cardille JA (2020). Remote sensing’s recent and future contributions to landscape ecology. Curr. Landsc. Ecol. Rep..

[22] Hooker J, Duveiller G, Cescatti A (2018). A global dataset of air temperature derived from satellite remote sensing and weather stations. Sci. Data..

[23] Huang R (2015). Mapping of daily mean air temperature in agricultural regions using daytime and nighttime land surface temperatures derived from TERRA and AQUA MODIS data. Remote Sens..

[24] Hoornweg D, Pope K (2017). Population predictions for the world’s largest cities in the 21st century. Environ. Urban..

[25] Ojeh V, Balogun A, Okhimamhe A (2016). Urban-rural temperature differences in Lagos. Climate..

[26] Babalola OS, Akinsanola AA (2016). Change detection in land surface temperature and land use land cover over Lagos Metropolis Nigeria. J. Remote Sens. GIS..

[27] Adeyeri OE, Akinsanola AA, Ishola KA (2017). Investigating surface urban heat island characteristics over Abuja, Nigeria: Relationship between land surface temperature and multiple vegetation indices. Remote Sens. Appl. Soc. Environ..

[28] Ayanlade A (2016). Variation in diurnal and seasonal urban land surface temperature: Landuse change impacts assessment over Lagos metropolitan city. Model. Earth Syst. Environ..

[29] Dissanayake D, Morimoto T, Murayama Y, Ranagalage M, Handayani HH (2019). Impact of urban surface characteristics and socio-economic variables on the spatial variation of land surface temperature in Lagos City, Nigeria. Sustainability.

[30] Bassett R, Young PJ, Blair GS, Samreen F, Simm W (2020). The megacity Lagos and three decades of urban heat island growth. J. Appl. Meteorol. Clim..

[31] Zhao J, Dong Y, Zhang M, Huang L (2020). Comparison of identifying land cover tempo-spatial changes using globcover and MCD12Q1 global land cover products. Arab. J. Geosci..

[32] Sulla-Menashe, D. & Friedl, M. A. User guide to collection 6 MODIS Land cover (MCD12Q1) product. *NASA EOSDIS Land Processes DAAC: Sioux Falls, SD, USA, 2018*. (2018).

[33] Liang D (2015). Evaluation of the consistency of MODIS land cover product (MCD12Q1) based on Chinese 30 M globeland30 datasets: A case study in Anhui Province, China. ISPRS Int. J. Geo-Inf..

[34] Zhou J, Chen Y, Wang J, Zhan W (2011). Maximum nighttime urban heat island (UHI) intensity simulation by integrating remotely sensed data and meteorological observations. IEEE J. Stars..

[35] Hertzberg M (2012). The night time radiative transport between the earth's surface, its atmosphere, and free space. Energy Environ..

[36] Shahmohamadi P, Che-Ani AI, Maulud KNA, Tawil NM, Abdullah NAG (2011). The impact of anthropogenic heat on formation of urban heat island and energy consumption balance. Urban Stud. Res..

[37] Liu Z (2017). Global and regional changes in exposure to extreme heat and the relative contributions of climate and population change. Sci. Rep..

[38] Shin J, Kang M, Kim KR (2022). Outdoor thermal stress changes in south korea: increasing inter-annual variability induced by different trends of heat and cold stresses. Sci. Total Environ..

[39] Simó G (2016). Landsat and local land surface temperatures in a heterogeneous terrain compared to MODIS values. Remote Sens..

[40] Lin L (2022). Validation and refinement of cropland data layer using a spatial-temporal decision tree algorithm. Sci. Data..

[41] Funk C (2019). Exploring trends in wet-season precipitation and drought indices in wet, humid and dry regions. Environ. Res. Lett..

[42] Lin L (2019). Building near-real-time MODIS data fusion workflow to support agricultural decision-making applications. IEEE.

